# Apology

**Published:** 1893-10

**Authors:** 


### EDITORIAL.

### APOLOGY.

It is an ill wind which blows nobody good, and our extreme regret at the necessary temporary suspension in the publication of the JOURNAL OF COMPARATIVE MEDICINE AND VETERINARY ARCHIVES has been somewhat lessened by the many letters which have assured us how much the JOURNAL is appreciated and valued by our readers.

A series of mishaps seemed to have combined to prevent the publication during the past few months. For the first, a worthless clerk was responsible, for losing some important copy and plates. Pressure of private business prevented the Editors from replacing the matter at once. As the July number was about ready for the printer a suit in Court over the title of the property which the JOURNAL rented for offices caused the editors to vacate, and all matter was for some time unavailable in a storage warehouse. Added to this, illness of the editors prevented the extra labor caused by the delays, which would have been necessary to issue a number. Our readers will be glad to hear that Dr. W. A. Conklin has recovered entirely from an almost fatal case of typhoid fever.

In resuming the publication, still under some difficulties, we tender to our readers our most sincere apologies for the intermission, and assure them that additional work and energy will be used to produce numbers which shall compensate for the missing ones.

### SUBSCRIPTIONS.

Subscriptions which have been paid in advance for 1893 will be credited until June, 1894.

Unpaid subscriptions commencing Jan. 1st, 1893, will be charged for six numbers to December, 1893, $1.50.

New subscriptions for the current year (January, February and March numbers can be supplied) will be $1.50.

Address all communications and payments :—JOURNAL COMPARATIVE MEDICINE AND VETERINARY ARCHIVES, 155 West 56th Street, New York.

